# Osteopontin and thrombospondin-1 play opposite roles in promoting tumor aggressiveness of primary resected non-small cell lung cancer

**DOI:** 10.1186/s12885-016-2541-5

**Published:** 2016-07-15

**Authors:** Mathieu Rouanne, Julien Adam, Aïcha Goubar, Angélique Robin, Caroline Ohana, Emilie Louvet, Jiemin Cormier, Olaf Mercier, Peter Dorfmüller, Soly Fattal, Vincent Thomas de Montpreville, Thierry Lebret, Philippe Dartevelle, Elie Fadel, Benjamin Besse, Ken André Olaussen, Christian Auclair, Jean-Charles Soria

**Affiliations:** INSERM Unit U981, Gustave Roussy Cancer Campus, 114, rue Edouard Vaillant, 94805 Villejuif, France; Université Paris Sud, Université Paris-Saclay, 94270 Le Kremlin-Bicêtre, France; CNRS UMR 8113, Ecole Normale Supérieure de Cachan, Cachan, France; Hôpital Foch, Université Versailles-Saint-Quentin-en-Yvelines, Université Paris-Saclay, 92150 Suresnes, France; Departement of Thoracic and Vascular Surgery, Centre Chirurgical Marie Lannelongue, Le Plessis-Robinson, France; Thoracic Multidisciplinary Committee, Institut d’Oncologie Thoracique, Le Plessis-Robinson, France; Department of Pathology, Centre Chirurgical Marie Lannelongue, Le Plessis-Robinson, France; Department of Biology, Centre Chirurgical Marie Lannelongue, Le Plessis-Robinson, France; Department of Cancer Medicine, Gustave Roussy Cancer Campus, Villejuif, France; Drug Development Department (DITEP: Département d’Innnovations Thérapeutiques et Essais Précoces), Gustave Roussy Cancer Campus, Villejuif, France

**Keywords:** Non-small cell lung cancer, Circulating biomarker, Thrombospondin-1, Osteopontin, Tumor microenvironment

## Abstract

**Background:**

Osteopontin (OPN) and thrombospondin-1 (TSP-1) are extracellular matrix proteins secreted by stromal and tumor cells. These proteins appear to have a key role in the tumor microenvironment for cancer development and metastasis. There is little information regarding the prognostic value of the combination of these two proteins in human cancers. Our aim was to clarify clinical significance and prognostic value of each circulating protein and their combination in primary resected non-small cell lung cancer (NSCLC) patients.

**Methods:**

We retrospectively reviewed 171 patients with NSCLC following curative intent surgery from January to December of 2012. Preoperative serums, demographics, clinical and pathological data and molecular profiling were analyzed. Pre-treatment OPN and TSP-1 serum levels were measured by ELISA. Tissue protein expression in primary tumor samples was determined by immunohistochemical analysis.

**Results:**

OPN and TSP-1 serum levels were inversely correlated with survival rates. For each 50 units increment of serum OPN, an increased risk of metastasis by 69 % (unadjusted HR 1.69, 95 % CI 1.12–2.56, *p* = 0.01) and an increased risk of death by 95 % (unadjusted HR 1.95, 95 % CI 1.15–3.32, *p* = 0.01) were observed. Conversely, for each 10 units increment in TSP-1, the risk of death was decreased by 85 % (unadjusted HR 0.15, 95 % CI 0.03–0.89; *p* = 0.04). No statistically significant correlation was found between TSP-1 serum level and distant metastasis-free survival (*p* = 0.2). On multivariate analysis, OPN and TSP-1 serum levels were independent prognostic factors of overall survival (HR 1.71, 95 % CI 1.04–2.82, *p* = 0.04 for an increase of 50 ng/mL in OPN; HR 0.18, 95 % CI 0.04–0.87, *p* = 0.03 for an increase of 10 ng/mL in TSP-1). In addition, the combination of OPN and TSP-1 serum levels remained an independent prognostic factor for overall survival (HR 1.31, 95 % CI 1.03–1.67, *p* = 0.03 for an increase of 6 ng/mL in OPN/TSP-1 ratio).

**Conclusions:**

Our results show that pre-treatment OPN and TSP-1 serum levels may reflect the aggressiveness of the tumor and might serve as prognostic markers in patients with primary resected NSCLC.

**Electronic supplementary material:**

The online version of this article (doi:10.1186/s12885-016-2541-5) contains supplementary material, which is available to authorized users.

## Background

Lung cancer, with non-small cell lung cancer (NSCLC) constituting 85 % of cases, remains the most prevalent and lethal cancer worldwide [1]. Surgery is the preferred initial treatment for patients with early stage disease and is correlated with a 5-year survival rate ranging from 70 % to 80 % in stage IA; and 30 % in stage IIIA disease [2]*.* With at least two-thirds of the relapses occurring in the 2–3 year period after initial resection [3], adjuvant platinum-based chemotherapy has been applied as the standard treatment for patients with early stage disease, and its application has shown an increase of 5-year survival rate by 4 ~ 5 % [4]. However, despite significant advances in the molecular mechanism that underpins NSCLC, the choice of adjuvant therapy is not guided by surrogate biomarkers [5]*.* Hence, there is a critical need to identify novel candidates for prognostic and predictive biomarkers.

Arguably, local microenvironment, alternatively known as niche, plays crucial roles in cancer progression that lead to dissemination of tumor cells into pre-metastatic niches of other organs [6]*.* Whereas genetic and epigenetic alterations in malignant cells have been extensively studied during the past decades, the role of tumor microenvironment has been largely overlooked [7]. It is suggested that tumor cells do not act alone but in close interaction with the extracellular matrix (ECM) and with non-genetically altered stromal cells [8]. Nevertheless, the dynamic process of dissemination and growth of cancer cells in metastatic sites remains unclear [9]. Members of matricellular proteins, such as osteopontin (OPN), thrombospondin (TSP), and SPARC (secreted protein acidic and rich in cysteine), exert their functions directly by either binding to cell surface receptors, or binding to other ECM proteins [10]. OPN and TSP-1 have important roles in a variety of biological processes, from cell adhesion and migration, to cell survival and proliferation and may interact with common target receptors (e.g., integrin αvβ3) [11]. However, such proteins appear to play contrasting roles in tumor progression and metastasis.

TSP-1 is a 450-kDa-homotrimeric protein composed of multiple functional domains that mediate cell-to-cell and cell-to-matrix interactions. The diverse biological activities of TSP-1 are mediated by its interaction with corresponding receptors such as integrins, CD36 and CD47, that are expressed on a variety of tumor and stromal cells [12]. Interestingly, TSP-1 was the first endogenous angiogenesis inhibitor to be identified [13]. Additionally, TSP-1 has been reported to inhibit tumorigenesis and metastasis in several tumor models [14]. Lack of TSP-1 has been associated with increased tumorigenesis; on the other hand, its over-expression or exogenous administration inhibits tumor formation and progression [15, 16]. To our knowledge, the role of TSP-1 in NSCLC pathogenesis has been poorly reported.

OPN is an extracellular matrix glycophosphoprotein consisting of three isoforms, OPN-a, OPN-b, and OPN-c, with molecular weights ranging from 41 to 75 kDa [17]. OPN contains several highly conserved structural elements including an integrin binding RGD domain, a calcium binding site and a heparin binding domain responsible for CD44 receptor binding [18]. OPN exerts its functions through direct binding to its receptors, which results in the activation of anti-apoptotic and pro-survival pathways, angiogenesis modulation, and ECM degradation [19]. OPN protumoral and prometastatic activities have been demonstrated in tumor animal models and in patients [20]. Although the over-expression of OPN is not unique to NSCLC, OPN appears to play a critical role in NSCLC carcinogenesis [21, 22]. In addition, clinical studies have shown that circulating OPN may serve as an important biomarker in early-stage and advanced cancer disease [23–26].

Due to contrasting effects on metastasis and angiogenesis, we hypothesized that the balance between circulating OPN and TSP-1 may impact patient survival. In addition, we supposed that the combination of circulating OPN and TSP-1 enhanced the prognostic value of each biomarker. The primary objective of this study was to determine the prognostic value of pre-treatment serums levels of OPN and TSP-1 and their combination in a cohort of primary resected NSCLC patients. The secondary objective was to assess the correlation between OPN and TSP-1 levels in serum and their expression in tumoral tissue.

## Methods

### Ethics statement

The Gustave Roussy Cancer Center and the Marie Lannelongue Institute Institutional Review Boards gave approval for this study. Written informed consent was obtained from included patients, and patient confidentiality was protected throughout the study.

### Patients and sample collection

Between January and December of 2012, 171 patients with primary NSCLC who underwent curatively intended surgical resection at Marie Lannelongue Hospital, France, were included in this study. Patients were staged according to the 7th edition of the IASLC/ATS/ERS classification [27–29] and the presence of adverse features such as visceral pleural invasion, lympho-vascular invasion and evidence of residual tumor at the resection margin were reviewed by a consultant histopathologist with expertise in lung cancer. Blood samples were obtained from each patient at baseline the day before surgery and centrifuged within 1 h of collection at 3600 × g for 10 min. Serums were stored at −20 °C until analysis was completed. Samples were then aliquoted and stored at −80 °C. Post-operative follow-ups included clinical and radiological examination (CT or conventional X-ray of the chest) at 3-month intervals for the first 2 years. Tumor recurrence and death during routine post-surgical follow-ups were recorded. First relapse was confirmed by pathologic diagnosis of the biopsy specimen.

### ELISA

OPN serum levels were measured using a commercially available enzyme test (Human OPN Quantikine ELISA Kit, R&D Systems, Minneapolis, MN, USA) and reported in ng/mL. All specimens were tested blinded and in duplicate. Serum samples from each patient were diluted 1:10 with calibrator Diluent RD5-24 and incubated in a micro titer plate coated with OPN antibody (Human OPN Quantikine ELISA Kit, R&D Systems, Minneapolis, MN, USA) for 2 h at room temperature (RT). After four washes, 200 μl of OPN conjugate (polyclonal antibody against OPN conjugated to horseradish peroxidase) was added to each well and incubated for 2 h at RT. Following four washes, 200 μl of substrate (hydrogen peroxide and chromogen) was added to each well and incubated for 30 min at RT. The absorbance of the samples was measured on a plate reader (Labsystems integrated EIA Management System) at 450 nm with wave length correction at 570 nm. Standard curve and sample values were calculated using Graph Pad Prism version 5.0 (GraphPad Software, Inc La Jolla, CA, USA). TSP-1 serum level was assessed using the same procedure, and each sample was assayed using Human TSP-1 Quantikine ELISA Kit (R&D Systems, Minneapolis, MN, USA) according to the manufacturer’s instructions. Controls were obtained from 20 healthy individuals (blood donors; 1:1 sex ratio).

### Immunohistochemical staining

For each patient, a pathologist selected one representative formalin-fixed, paraffin-embedded (FFPE) tumor block of the primary tumor. Immunohistochemistry was performed on 3 μm thick whole sections using a validated standard protocol on a Ventana Discovery Ultra autostainer (Ventana Medical Systems, Roche Tissue Diagnostics, Tucson, AZ, USA). After deparaffinization and antigen retrieval in CC1 buffer for 32 min at 98 °C, sections were incubated with either a primary goat polyclonal antibody (Santa Cruz Biothechnology; clone; concentration; dilution 1:100) against human OPN or a primary mouse monoclonal antibody (Santa Cruz Biothechnology; clone A 6.1; concentration; dilution 1:100) against TSP-1 for 1 h at room temperature. Amplification was achieved using an UltraView anti-rabbit HRP kit and with diaminobenzidine as chromogen. Slides were counterstained with hematoxylin. A pathologist who was blinded to the clinical data scored the immunohistochemical staining intensity. Two different scores were used to assess OPN and TSP-1 tissue expression because of the type of staining. OPN staining on whole slides was heterogeneous within different areas in many tumors while TSP-1 staining was much more homogeneous. H-score including both intensity of staining and percentage of stained tumor cells was used as an exploratory evaluation for OPN IHC staining. Thus, the percentage of positive tumor cells was multiplied by the staining intensity of tumor cell to obtain a final semi quantitative H score (0–300). For TSP-1, a scoring based only on staining intensity (0 to 3) was used, similarly to intensity scoring in the H-score. Then, the intensity of the staining in tumor cells was scored using a semi-quantitative scale: 0 (negative), 1 (weak), 2 (moderate), and 3 (strong). The intensity of the tumor infiltrating immune cells expressing each marker was also scored using a semi-quantitative scale from 0 (no infiltrate), 1 (mild infiltration), 2 (moderate infiltration) to 3 (dense infiltration). Image acquisition was performed with a Virtual Slides microscope VS120-SL (Olympus, Tokyo, Japan), 20X air objective (0.75 NA).

### Molecular profiling

Systematic molecular analysis was performed on each tumor sample at the Genomics Platform (Gustave Roussy Institute, Villejuif, France) to identify EGFR, HER2, KRAS, BRAF, and PI3K mutation status. DNA was extracted from sections of FFPE tissues. Histological examination showed that more than 30 % of the cells were tumor cells. Genomic DNA was extracted from 4 × 10 μm thick FFPE block sections using the DNeasy Blood and tissue kit (QIAGEN, Hilden, Germany) according to the manufacturer’s instructions. All coding sequences of exons 18 to 21 of EGFR gene (GeneBank NM005228.3); exons 2 and 3 of Kirsten rat sarcoma viral oncogene (K-RAS) gene (NM033360-2); exon 15 of v-Raf murine sarcoma viral oncogene homolog B1 (BRAF) gene (NM004333.4); exons 10 and 21 of phosphatidylinositol-4,5-bisphosphate 3-kinase, catalytic subunit alpha (PIK3CA) gene (NM006218.2); and exon 20 of human epidermal growth factor receptor-2 (HER2) gene (NM 004448.2) were analyzed. Sanger direct sequencing was performed using Big Dye Terminator Cycle Sequencing Kit (Applied Biosystems, Foster City, CA, USA) after polymerase chain reaction amplification of targeted exons. Sequencing reactions were analyzed on 48-capillary 3730 DNA Analyzer (Applied Biosystems, Foster City, CA, USA). Sequence reading and alignment were performed with Seq Scape software (Applied Biosystems, Forster City, CA, USA). Detected mutations were confirmed by an independent *t* test. ALK rearrangement status was determined by fluorescent in situ hybridization assay on tumor tissues using Dako Pre-treatment Kit (Dako) and Vysis ALK Break Apart Rearrangement Probe Kit (Abott Molecular Inc., Des Plaines, IL, USA) according to protocols described previously [30].

### Statistical analyses

Statistical analysis was carried out using R [31]. Two survival end points were evaluated: (i) distant metastasis-free survival (DMFS), defined as the time interval between surgery and date of distant relapse or death, and (ii) overall survival (OS), defined as the time interval between surgery and death. Patients who were alive (OS) or without distant relapse (DMFS) were censored at the date of last contact. The prognostic values of serum OPN and TSP-1 were first tested as individual variables. Then we assessed the significance of the combination of these two variables. Hazard ratios (HR) and 95 % confidence interval (95 % CI) were calculated with the Cox proportional hazard regression model. Independent clinico-pathological variables were first analysed with univariate analysis. Variables shown in the univariate analysis that were significantly associated with DMFS and OS (with *p* < 0.2) were further analyzed in a multivariate cox proportional hazards regression model. Only the results of those variables that were significantly associated with the DMFS and OS (*p* < 0.05) using the stepwise variable selection method were reported. The required assumptions of proportionality in the multivariate survival analysis were checked graphically and by Schoenfeld’s test. DMFS and OS curves were estimated using the Kaplan–Meier method and values between groups were compared using the log-rank test. The association between OPN and TSP-1 serum levels and clinico-pathological variables were tested using Mann–Whitney *U*-test. Percentage, median, and range were reported to statistically describe the data. The correlation between OPN serum level and OPN tissue expression level was calculated using the Spearman method. All the statistical tests were two sided with a *p* value of 0.05.

## Results

### Clinicopathologic and molecular characterisitcs of patients

Baseline characteristics of the patients are reported in Table 1. A total of 171 subjects were identified with a sex ratio of 2:1 (male: female) and a median age of 62 years (range 40–93). Non-smokers represented 16 % of the population. Primary lung adenocarcinoma was the most prevalent histotype (63 %), followed by squamous cell carcinoma (23 %). Tumor EGFR mutation, KRAS mutation and ALK rearrangement status were available for 124 (72.5 %), 126 (74 %), and 167 (98 %) patients, respectively. Pathological examination classified 92 % of the patients as having stage I to IIIA, including 47 % stage I, 21 % stage II, and 24 % stage IIIA. Thirteen patients (8 %) were classified as having stage IIIB. The majority (74 %) of resected specimen tumor sizes were lower than 5.0 cm in greatest dimension (range 0.8–11.0). All patients underwent mediastinal lymph node sampling or dissection. Lymphatic diffusion to N1, N2 and N3 nodes was present in 16 %, 22 %, and 1 % of the patients, respectively. No patient received neoadjuvant chemotherapy or radiotherapy. According to clinical criteria, a total of 48 (29 %) patients received adjuvant systemic platinum-based chemotherapy; among which combined adjuvant radio-chemotherapy was initiated in eight subjects (5 %).

**Table 1:** Serum concentration of osteopontin (OPN) and thrombospondin-1 (TSP-1) according to baseline patients characteristics

	No (%)	OPN serum level		TSP-1 serum level	
		Median (range)	*p* value	Median (range)	*p* value
Population					
Controls	20	8.8 (4–46)		31 (0–12060)	
Patients	171 (100)	27.6 (7–191)	<1.10^e-5^	14520 (2946–30940)	<1.10^e-5^
Age, years (range)					
<65	138 (81)	26.3 (7–89)		15057 (3979–30938)	
≥65	33 (19)	32.1 (9–191)	0.07	13171 (2946–22011)	0.01
Sex					
Female	62 (36)	26.6 (7–89)		14910 (2946–30938)	
Male	109 (64)	28.2 (8–191)	0.17	13980 (3381–26077)	0.31
Smoking history					
Current	87 (51)	27.9 (7–88)		15062 (2946–30938)	
Former	56 (33)	27.9 (7–191)		13171 (3381–26077)	
Never	27 (16)	25.0 (9–89)	0.85	14762 (5564–20468)	0.17
p TNM					
IA	40 (23)	23.3 (11–89)		15270 (3381–25112)	
IB	41 (24)	24.5 (7–87)		13120 (4899–26906)	
IIA	21 (12)	26.7 (8–88)		12928 (7047–24731)	
IIB	16 (9)	30.1 (9–78)		14440 (9043–23813)	
IIIA	40 (24)	33.4 (8–148)		13639 (5450–30938)	
IIIB	5 (3)	37.5 (17–191)		14816 (12862–20606)	
IV	8 (5)	26.4 (9–64)	0.17	16332 (2946–22299)	0.75
Stage					
I-II-IIIA	158 (92)	27.4 (7–148)		14423 (3381–30938)	
IIIB	13 (8)	29.6 (9–191)	0.75	16324 (2946–22299)	0.23
Tumor size					
<5 cm	125 (74)	23.3 (7–89)		14107 (2946–30938)	
≥5cm	43 (26)	34.6 (9–191)	<1.10^e-5^	15057 (5450.5-23813)	0.53
Type of resection					
Wedge	22 (14)	26.11 (8–89)		13403 (2946–22299)	
Lobectomy	123 (77)	26.8 (7–148)		15062.(3381–26906)	
Bi-lobectomy	6 (4)	31.2 (12–191)		15804(11052–21302)	
Pneumonectomy	9 (6)	38.3 (9–85)	0.17	10526 (5929–30938)	0.07
Resection margins					
Clear	165 (98)	27.2 (7–191)		14519 (2946–30938)	
Involved	4 (2)	54.5 (9–75)	0.26	15410 (12605–22011)	0.43
Histologic subtype					
Adenocarcinoma	108 (63)	25.3 (7–148)		14799 (2946–26906)	
Squamous cell carcinoma	40 (23)	32.8 (8–88)		13032 (5327–30938)	
Large cell carcinoma	8 (5)	36.3 (21–72)		15720 (12480–22299)	
Other	15 (9)	29.6 (9–191)	0.06	12605 (5564–24731)	0.29
Lympho-vasc. invasion					
Yes	42 (26)	28.4 (9–191)		14202 (2946–24731)	
No	123 (75)	27.6 (7–148)	0.47	14567 (3979–30938)	0.75
Pleural invasion					
Yes	74 (46)	28.3 (7–191)		13811 (2946–26077)	
No	86 (54)	26.9 (8–89)	0.36	15247 (3381–30938)	0.27
Adjuvant treatment					
Yes	48 (29)	28.1 (7–191)		14608 (5327–24731)	
No	120 (71)	26.9 (8–148)	0.69	14543 (2946–30938]	0.66
EGFR status					
Wild-type	112 (90)	26.8 (7–191)		13752 (2946–26906)	
Mutation	12 (10)	24.6 (10–38)	0.37	15984 (10111–22165)	0.23
KRAS status					
Wild-type	78 (62)	27.4 (7–188)		13171 (2946–26906)	
Mutation	48 (38)	24.7 (7–191)	0.37	15323 (5327–25112)	0.08
ALK rearrangement					
Yes	3 (2)	29.6 (22–64)		19801 (14519–22299)	
No	164 (98)	27.2 (7–191)	0.50	14440 (2946–30938)	0.13

### Pre-treatment OPN and TSP-1 serum levels

Baseline serums were analyzed in 171 patients before primary tumor removal. OPN serum levels ranged from 7 to 191 ng/ml, with a median value of 27.6 ng/ml; TSP-1 serum levels ranged from 2946 to 30940 ng/ml, with a median value of 14520 ng/ml. From the control group of healthy individuals (*n* = 20), the median OPN and TSP-1 levels were 8.8 ng/ml [4–45], and 31 ng/ml (0–12060), respectively. The difference in serum levels between patients and donors was statistically significant for both OPN and TSP-1 (*p* < 0.005) (Additional file 1). The association between clinicopathologic parameters and serum levels is presented in Table 1 and Additional file 2. Patients over 65 years old were more likely to have higher levels of OPN (32.1 *vs* 26.3 ng/mL, respectively, *p* = 0.07) and significantly lower TSP-1 serum levels compared to younger patients (13171 *vs* 15057 ng/mL, respectively, *p* = 0.01). No difference was found in serum levels with regard to smoking history. We noticed an increase of OPN serum level from stage I to IIIB. However, no significant difference was observed for either OPN or TSP-1 serum levels when the patient population was classified into pTNM stage. OPN serum level was significantly lower in patients with tumor size <5 cm than in those with size ≥5 cm (*p* < 0.0001). In regard to pathologic features, neither OPN nor TSP-1 serum levels was statistically associated with pleural involvement or lympho-vascular invasion. OPN serum level was lower in lung adenocarcinoma with borderline significance (*p* = 0.06) whereas no statistical association was found regarding pathological status and TSP-1. Small differences in serum levels among the molecular status were observed, however the difference did not exhibit statistical significance. Patients with KRAS mutation tend to have higher TSP-1 serum levels compared to those with KRAS wild-type (15323 *vs*13171, respectively, *p* = 0.08).

### Prognostic value of pre-treatment OPN and TSP-1 serum levels

Median follow-up was 26 months (6–34 months). Univariate and multivariate analysis evaluating the association between survival and individual or combinatorial markers are reported in Table 2 (OS) and Table 3 (DMFS). OPN serum levels as continuous variables were significantly associated with both DMFS and OS in univariate analysis (Table 2). For each 50 units increment of serum OPN, an increased risk of metastasis by 69 % (unadjusted HR 1.69, 95 % CI 1.12–2.56, *p* = 0.01) and an increased risk of death by 95 % (unadjusted HR 1.95, 95 % CI 1.15–3.32, *p* = 0.01) were observed. Conversely, TSP-1 serum levels as continuous variables were inversely correlated with OS in univariate analysis. For each 10 units increment in TSP-1, the risk of death was decreased by 85 % (unadjusted HR 0.15, 95 % CI 0.03–0.89; *p* = 0.04). No statistically significant correlation was found between TSP-1 serum level and DMFS (*p* = 0.23). The combination of OPN and TSP-1 serum levels as continuous variable, measured as the ratio of OPN to log 10 transformation of TSP-1, was significantly correlated with both DMFS and OS in univariate analysis. For an increment of 6 ng/mL in this ratio, a 30 % increased risk of metastasis (unadjusted HR 1.3, 95 % CI 1.06–1.59, *p* = 0.01) and 40 % increased risk of death (unadjusted HR 1.4, 95 % CI 1.08–1.81, *p* = 0.01) were observed. In stage I to IIIA, absence of pleural involvement and lympho-vascular invasion were also associated with better clinical outcome in univariate analysis (Tables 2 and 3).

**Table 2:** Univariate and multivariate analysis of clinicopathological factors with serum osteopontin level (A), serum thrombospondin-1 level (B), serum OPN/TSP-1 ratio (C) for overall survival

		Univariate analysis			Multivariate analysis		
		HR	95 % CI	*p* value	HR	95 % CI	*p* value
A. Factors associated with serum osteopontin level for overall survival							
Osteopontin	Serum level^a^	1.95	1.15–3.32	0.01	1.71	1.04–2.82	0.04
Stage	I to IIIA *vs* IIIB	0.26	0.11–0.61	0.002	0.29	1.04–0.68	0.01
Pleural involvement	Positive *vs* Negative	1.94	0.93–4.04	0.08	1.11	0.66–1.57	0.73
Lympho-vascular invasion	Positive *vs* Negative	2.47	1.21–5.03	0.01	1.27	0.56–2.85	0.57
Adjuvant treatment	No *vs* Yes	1.32	0.63–2.73	0.51	–	–	–
Age	≥65 *vs* <65	1.61	0.75–3.46	0.24	–	–	–
Gender	Male *vs* female	1.14	0.56–2.34	0.73	–	–	–
Smoking history	Smoker *vs* non-smoker	1.14	0.44–2.96	0.80	–	–	–
B. Factors associated with serum thrombospondin-1 level for overall survival							
Thrombospondin-1	Serum level^b^	0.15	0.03–0.89	0.04	0.18	0.04–0.87	0.03
Stage	I to IIIA *vs* IIIB	0.26	0.11–0.61	0.002	0.26	0.11–0.61	0.002
Pleural involvement	Positive *vs* Negative	1.94	0.93–4.04	0.08	1.13	0.36–1.37	0.69
Lympho-vascular invasion	Positive *vs* Negative	2.47	1.21–5.03	0.01	1.28	0.58–2.84	0.54
Adjuvant treatment	No *vs* Yes	1.32	0.63–2.73	0.51	–	–	–
Age	≥65 *vs* <65	1.61	0.75–3.46	0.24	–	–	–
Gender	Male *vs* female	1.14	0.56–2.34	0.73	–	–	–
Smoking history	Smoker *vs* non-smoker	1.14	0.44–2.96	0.80	–	–	–
C. Factors associated with serum osteopontin/thrombospondin-1 ratio for overall survival							
OPN/TSP1	Ratio^c^	1.40	1.08–1.81	0.01	1.31	1.03–1.67	0.03
Stage	I to IIIA *vs* IIIB	0.26	0.11–0.61	0.002	0.29	0.12–0.68	0.01
Pleural involvement	Positive *vs* Negative	1.94	0.93–4.04	0.08	0.75	0.24–0.97	0.38
Lympho-vascular invasion	Positive *vs* Negative	2.47	1.21–5.03	0.01	0.62	0.23–0.89	0.27
Adjuvant treatment	No *vs* Yes	1.32	0.63–2.73	0.51	–	–	–
Age	≥65 *vs* <65	1.61	0.75–3.46	0.24	–	–	–
Gender	Male *vs* female	1.14	0.56–2.34	0.73	–	–	–
Smoking history	Smoker *vs* non-smoker	1.14	0.44–2.96	0.80	–	–	–

*HR* Hazard Ratio, *95 % CI* 95 % Confidence interval

^a^for an increment in OPN of 50 ng/mL

^b^for an increment in TSP-1 of 10 ng/mL in log10 scale

^c^for an increment of 6 ng/mL in the ratio OPN/log10 (TSP1)

**Table 3:** Univariate and multivariate analysis of clinicopathological factors with serum osteopontin level (A), serum thrombospondin-1 level (B), serum OPN/TSP-1 ratio (C) for distant metastasis-free survival (DMFS)

		Univariate analysis			Multivariate analysis		
		HR	95 % CI	*p* value	HR	95 % CI	*p* value
A. Factors associated with serum osteopontin level for distant metastasis-free survival							
Osteopontin	Serum level^a^	1.69	1.12–2.56	0.01	1.35	0.93–1.97	0.12
Stage	I to IIIA *vs* IIIB	0.23	0.12–0.44	<0.0001	0.31	0.15–0.63	0.001
Pleural involvement	Positive *vs* Negative	2.58	1.50–4.43	<0.001	1.91	1.07–3.40	0.03
Lympho-vascular invasion	Positive *vs* Negative	2.33	1.37–3.87	0.002	1.80	1.02–3.17	0.04
Adjuvant treatment	No *vs* Yes	1.20	0.70–2.04	0.54	–	–	–
Age	≥65 *vs* <65	1.05	0.56–1.97	0.91	–	–	–
Gender	Male *vs* female	0.97	0.59–1.61	0.92	–	–	–
Smoking history	Smoker *vs* non-smoker	1	0.51–1.97	1	–	–	–
B. Factors associated with serum thrombospondin-1 level for distant metastasis-free survival							
Thrombospondin-1	Serum level^b^	0.39	0.10–1.45	0.23	–	–	–
Stage	I to IIIA *vs* IIIB	0.23	0.12–0.44	<0.0001	0.29	0.14–0.58	0.001
Pleural involvement	Positive *vs* Negative	2.58	1.50–4.43	<0.001	1.91	1.07–3.41	0.03
Lympho-vascular invasion	Positive *vs* Negative	2.33	1.37–3.87	0.002	1.79	1.01–3.14	0.04
Adjuvant treatment	No *vs* Yes	1.20	0.70–2.04	0.54	–	–	–
Age	≥65 *vs* <65	1.05	0.56–1.97	0.91	–	–	–
Gender	Male *vs* female	0.97	0.59–1.61	0.92	–	–	–
Smoking history	Smoker *vs* non-smoker	1	0.51–1.97	1	–	–	–
C. Factors associated with serum osteopontin/thrombospondin-1 ratio for distant metastasis-free survival							
OPN/TSP1	Ratio^c^	1.31	1.06–1.59	0.01	1.16	0.97–1.40	0.11
Stage	I to IIIA *vs* IIIB	0.23	0.12–0.44	<0.0001	0.31	0.15–0.63	0.001
Pleural involvement	Positive *vs* Negative	2.58	1.50–4.43	<0.001	1.90	1.07–3.40	0.03
Lympho-vascular invasion	Positive *vs* Negative	2.33	1.37–3.87	0.002	1.80	1.02–3.17	0.04
Adjuvant treatment	No *vs* Yes	1.20	0.70–2.04	0.54	–	–	–
Age	≥65 *vs* <65	1.05	0.56–1.97	0.91	–	–	–
Gender	Male *vs* female	0.97	0.59–1.61	0.92	–	–	–
Smoking history	Smoker *vs* non-smoker	1	0.51–1.97	1	–	–	–

*HR* Hazard Ratio, *95 % CI* 95 % Confidence interval

^a^for an increment in OPN of 50 ng/mL

^b^for an increment in TSP-1 of 10 ng/mL in log10 scale

^c^for an increment of 6 ng/mL in the ratio OPN/log10 (TSP1)

**Fig. 1:**
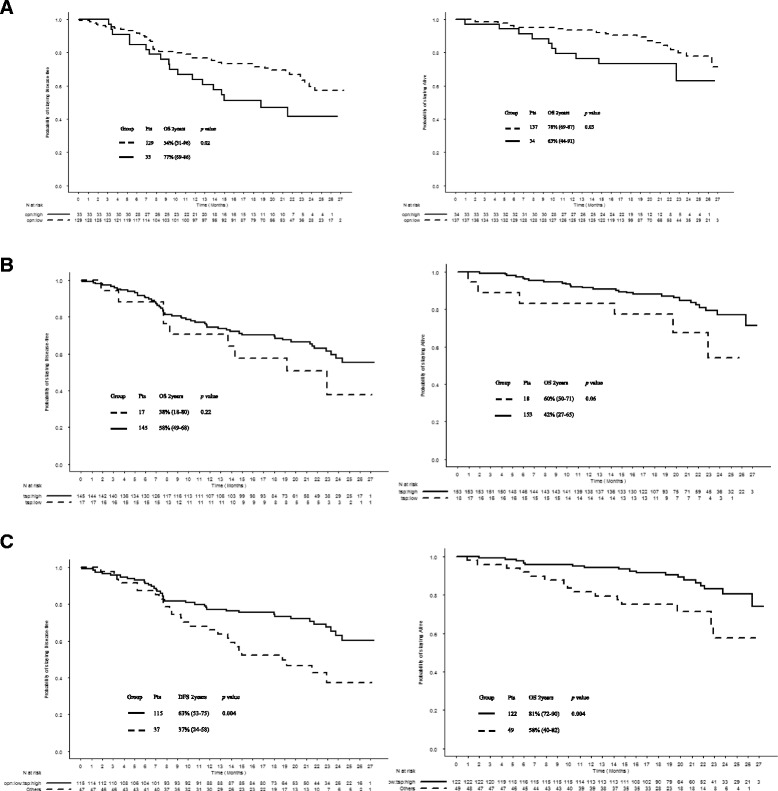
Estimated Kaplan-Meier curves for distant metastasis-free and overall survival categorized by the serum level of (**a**) osteopontin, (**b**) thrombospondin-1 and (**c**) their combination. The osteopontin high group was defined as the top 20 % patient population with the highest values in osteopontin level and the osteopontin low group represented the rest of the population. Thrombospondin-1 low group was defined as the top 10 % patient population with the lowest values in thrombospondin-1 level and thrombospondin-1 high group represented the rest of the population

Three separate multivariate analyses that included prognostic clinical parameters and each of the three following variables: OPN and TSP-1 serum levels, and their combination were performed. As reported in Table 2, both OPN and TSP-1 serum levels as well as their combination maintained a strong prognostic value in the multivariate model for OS (HR 1.71, 95 % CI 1.04–2.82, *p* = 0.04; HR 0.18, 95 % CI 0.04–0.87, *p* = 0.03 and HR 1.31, 95 % CI 1.03–1.67, *p* = 0.03, for OPN, TSP-1 and combination, respectively). No correlation was found between these variables and DMFS (*p* > 0.05). The other independent prognostic factor was pathological stage. The relationship between the three markers and survival were illustrated using Kaplan-Meier survival curves represented in Fig. 1.

### Tumor tissue expression of OPN and TSP-1

**Fig. 2:**
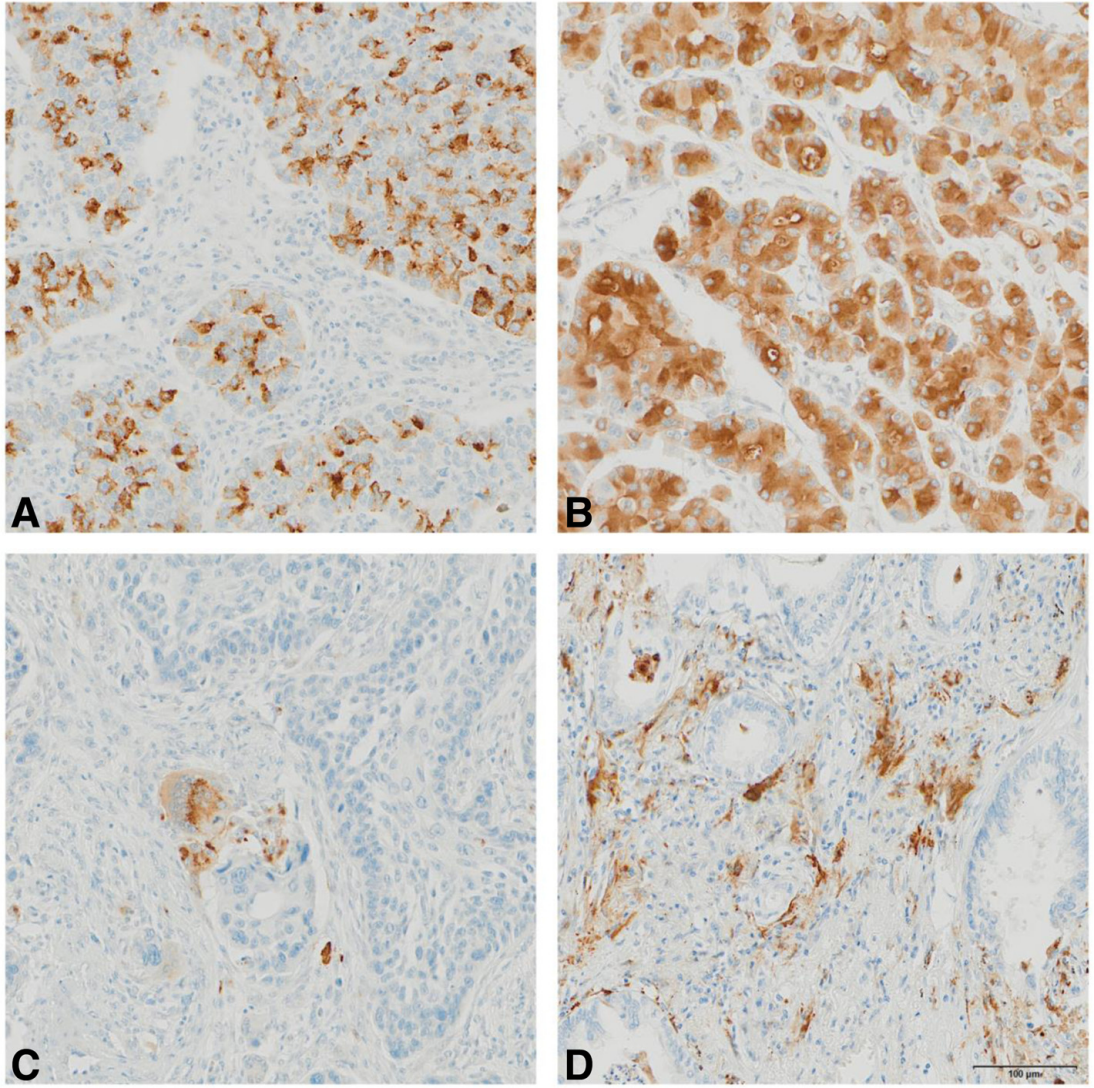
Representative immunohistochemical staining showing positive (**a** = moderate, **b** = strong), negative (**c**) osteopontin tumor cells expression and positive (**d**) stroma staining

**Fig. 3:**
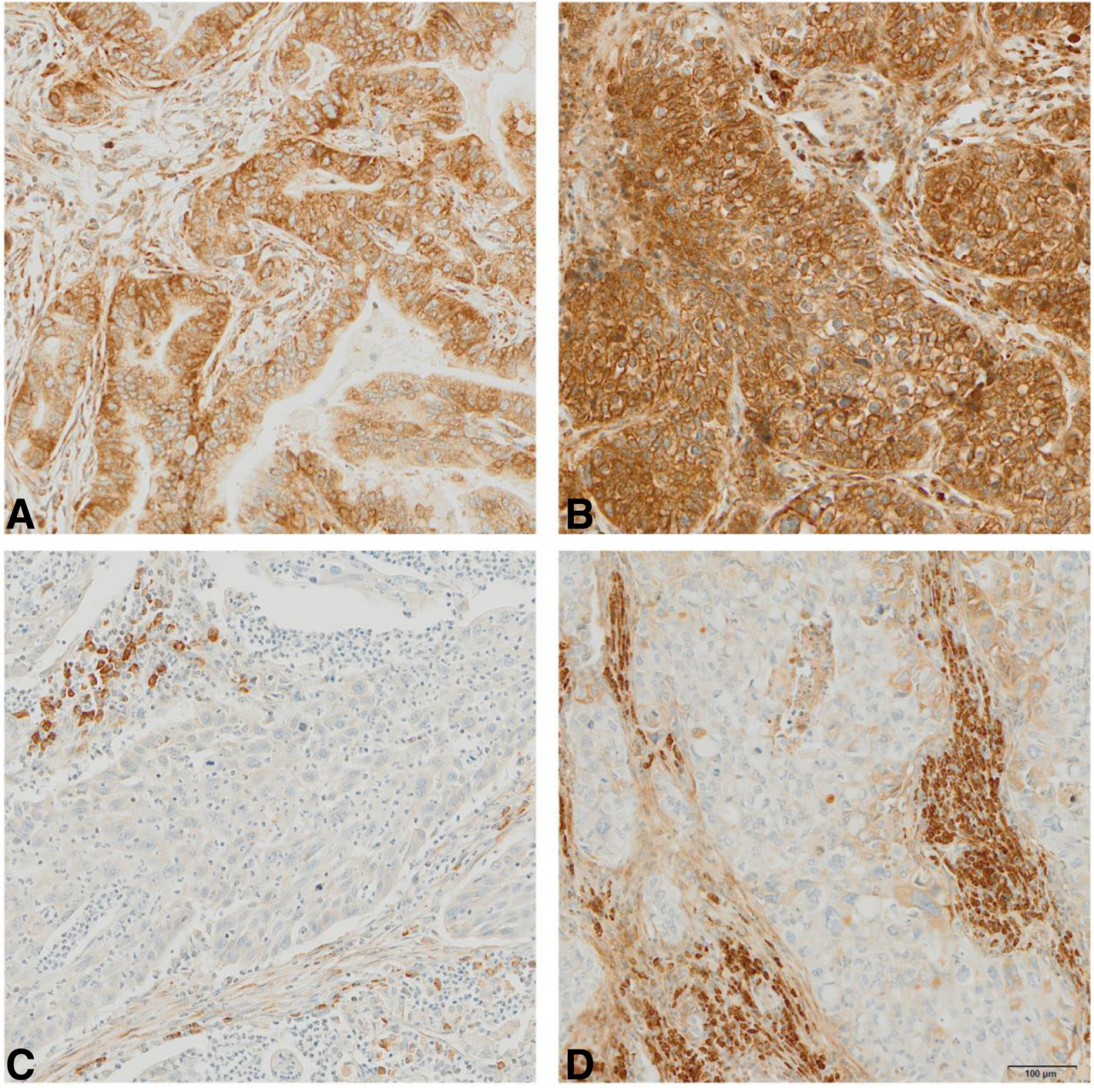
Representative immunohistochemical staining showing positive (**a** = moderate, **b** = strong), negative (**c**) thrombospondin-1 tumor cells expression and positive (**d**) stroma staining

An overview of the immunohistochemical expression patterns of OPN and TSP-1 in tumor cells and stroma is presented in Figs. 2 and 3. The staining intensities of OPN and TSP-1 were positive in the tumor cells and the tumor stroma with heterogeneous expression. Thirty eight percent of cases showed strong cytoplasmic staining of OPN in tumor cells, while 26 % of cases were moderate, and 36 % negative (Fig. 2a,b,c). Patients with negative stroma staining (score = 0) were compared to the other cases (score = 1 or 2) (Fig. 2d). Sixty-seven percent of the samples were stained positive for TSP-1, with 37 % of cases showing strong staining and 30 % displaying moderate staining (Fig. 3a and b). In the tumor stroma, some immune cells were stained, prominently macrophages for OPN (Fig. 2d), and lymphocytes and plasmocytes for TSP-1 (Fig. 3d). There was no significant association between TSP-1 serum level and TSP-1 tissue expression level. OPN serum level and OPN tissue expression level were weakly correlated (Spearman coefficient correlation = 0.26) (Tables 2 and 3).

## Discussion

In this study, we assessed the prognostic values of circulating OPN and TSP-1 in a cohort of 171 primary operable NSCLC patients. We suggested that pre-treatment serum levels of OPN and TSP-1 might have potential prognostic value in primary resected NSCLC patients. Metastases represent the final step of a complex process initiated by tumor cells from primary site to distant sites. It is now believed that only a very small subset of circulating tumor cells form macrometastasis (0.02 %); suggesting that microenvironment of distant sites are generally inhospitable for tumor cell growth [6, 7]. Recent studies reported that tumor and stroma cells from the primary site secrete soluble proteins including extracellular matrix proteins and growth factors to prepare metastatic niche [32, 33]. Herein, we hypothesized that the balance between the antiangiogenic effect from TSP-1 and anti-apoptotic activity from OPN may create an enriched tumor microenvironment that enhance cell seeding and metastasis development.

Several studies have emphasized the role of OPN in tumorigenesis, progression and metastatic dissemination in NSCLC [34–36]. It is well known that circulating OPN levels correlate with clinical outcomes in several types of advanced human cancers [37–39]. Until the recent few years, clinical implications of circulating OPN in patients with NSCLC were not well established. Recently, three clinical studies found that OPN plasma levels were significantly associated with progression-free survival and overall survival in patients with advanced NSCLC who received either chemotherapy or radiotherapy [23, 25, 40]. Takenaka et al. [41] found that OPN serum level was an unfavorable prognostic predictor not only for patients with advanced NSCLC but also for patients with stage I disease. Similarly, we noted that an increase in serum OPN level was associated with poor prognosis according to survival outcomes in primary resected NSCLC patients.

In our study, we reported a median serum OPN level of 27.6 ng/ml (7–191 ng/ml), which is comparable to results from previous series in serum [41] or plasma [26]. The significant difference between OPN serum levels in healthy individuals *versus* NSCLC patients promotes the potential diagnostic relevance of OPN as a biomarker for NSCLC. Furthermore, we found that OPN serum levels increased significantly according to tumor size, suggesting that its production by tumor cells prevailed. Interestingly, Blasberg et al. [26] reported that baseline OPN plasma levels were reduced after primary resection of NSCLC tumor. They also demonstrated that OPN plasma levels had risen when the patients relapsed. Contrary to other reports, no correlation was found between OPN tumor tissue expression in primary tumor and clinical outcomes. In our study, immunohistochemical staining was performed on whole tumor sections instead of tissue microarray, which avoided bias interpretation due to intratumoral heterogeneity. Other possible explanations for the contradicting results could be that different antibodies, immunohistochemical staining techniques and scoring systems were used. Lack of correlation between serum levels and tissue expression may be explained by the variability of OPN production from normal and tumor cells.

Our study showed that an increase in serum TSP-1 level was associated with good prognosis according to survival analysis. Arguably, several studies have reported the ability of TSP-1 to inhibit tumor angiogenesis. Yamaguchi et al. demonstrated that reduced expression of TSP-1 was correlated with poor prognosis in NSCLC patients [42]. Indeed, strong evidence suggests an inhibitory role of TSP-1 in cancer cell proliferation and metastasis [43]. Inhibitory effect of TSP-1 in cancer cell proliferation and metastasis comes from studies in human lung cancer cell lines, whereas an inverse correlation has been reported between TSP-1 messenger RNA, protein expression and malignant progression [44]. Furthermore, transfection studies in breast cancer cell lines have demonstrated that production of TSP-1 in tumor cell exerts an inhibitory effect on tumor progression [45].

We also found a median TSP-1 serum level of 14520 ng/ml (2946–30940 ng/ml), which was much higher compare to OPN serum levels in the same cohort. Significant difference was observed between patients and controls (*p* < 0.001). Compared to OPN, we found an inverse correlation between TSP-1 serum level and clinical outcomes, suggesting a protective role for TSP-1. Surprisingly, we found a statistically significant association between age and TSP-1 serum level, suggesting that people under 65 years old secreted more TSP-1. Nevertheless, age was not identified as prognostic factor in univariate and multivariate analyses. Similarly, tumor expression was heterogeneous with variable staining intensity among tumor and stroma cells, and no correlation was observed with patients outcomes. Secretion from a variety of normal and tumor cell types may explain the absence of correlation between serum levels and tissue expression.

To our knowledge, this is the first study investigating the prognostic value of circulating OPN and TSP-1 in primary resected NSCLC patients. One of the major strength of our study was to assess simultaneously pre-treatment circulating OPN and TSP-1 in the same cohort of NSCLC patients who underwent curative intent surgery. Hence, combining OPN and TSP-1 serum levels may enhance the prognostic value of each biomarker and more accurately reflect the aggressiveness of the tumor. Nevertheless, our study suffers from several limitations. First, this is a retrospective serie with a small sample size and a statistical power limited by short-term follow-up. In addition, the potential use of tyrosine kinase inhibitors in 12 (7 %) patients with targetable genomic alterations (i.e. activating EGFR mutations and ALK translocation) was not assessed in the univariate and multivariate analysis for overall survival. Second, biology of matricellular proteins is complex. Indeed, intracellular forms of OPN and TSP-1 probably mediate different metastatic activity than exogenous proteins. To understand the role matricellular proteins play within the tumor microenvironment, it is also important to decipher OPN and TSP-1 interaction networks occurring between tumor cells and stroma. Finally, better understanding of cell signaling mechanisms induced by OPN and TSP-1 is essential to deeply explore the cancer pathways activated among tumor cells.

## Conclusion

Our results show that pre-treatment OPN and TSP-1 serum levels may reflect the aggressiveness of the tumor and might serve as prognostic markers in patients with primary resected NSCLC. This study provides further support of the importance of tumor microenvironment in the biology of NSCLC. Undoubtedly, better understanding of the cellular constituents of tumors and interactions between malignant and stromal cells may help develop next generation innovative cancer therapeutics.

## Abbreviations

EGFR, epidermal growth factor; ELISA, enzyme-linked immunosorbent assay; FFPE, formalin fixed paraffin embedded; HER2, human epidermal growth factor receptor-2; IASLC/ATS/ERS, International Association for the Study of Lung Cancer/the American Thoracic Society/the European Respiratory Society; K-RAS, Kirsten rat sarcoma viral oncogene; NSCLC, non-small cell lung cancer; OPN, osteopontin; PIK3CA, phosphatidylinositol-4,5-bisphosphate 3-kinase; TSP-1, thrombospondin-1.

## Additional files

Additional file 1: Figure S1. (A). Graphical representation of the ELISA data for OPN serum levels in healthy donors (*n* = 20) and population study (*n* = 171). Figure S1 (B). Graphical representation of the ELISA data for TSP-1 serum levels in healthy donors (*n* = 20) and population study (*n* = 171). (DOCX 154 kb) Additional file 2: Dataset supporting the conclusions of this article. (XLSX 58 kb)

## References

[1] Siegel R, Ma J, Zou Z, Jemal A (2014). Cancer statistics, 2014. CA Cancer J Clin.

[2] Pignon JP, Tribodet H, Scagliotti GV (2008). Lung adjuvant cisplatin evaluation: a pooled analysis by the LACE Collaborative Group. J Clin Oncol..

[3] Demicheli R, Fornili M, Ambrogi F (2012). Recurrence dynamics for non-small-cell lung cancer: effect of surgery on the development of metastases. J Thorac Oncol..

[4] Arriagada R, Auperin A, The NSCLC Meta-analyses Collaborative Group (2010). Adjuvant chemotherapy, with or without postoperative radiotherapy, in operable non-small-cell lung cancer: two meta-analyses of individual patient data. Lancet.

[5] Friboulet L, Olaussen KA, Pignon JP (2013). ERCC1 isoform expression and DNA repair in non-small-cell lung cancer. N Engl J Med.

[6] Wong GS, Rustgi K (2013). Matricellular proteins: priming the tumour microenvironment for cancer development and metastasis. Br J Cancer.

[7] Swartz MA, Iida N, Roberts EW (2012). Tumor Microenvironment Complexity: Emerging Roles in Cancer Therapy. Cancer Res..

[8] Hanahan D, Coussens LM (2012). Accessories to the crime: functions of cells recruited to the tumor microenvironment. Cancer Cell..

[9] Chambers AF, Groom AC, MacDonald IC (2002). Dissemination and growth of cancer cells in metastatic sites. Nat Rev Cancer.

[10] Chiodoni C, Colombo MP, Sangaletti S (2010). Matricellular proteins: from homeostasis to inflammation, cancer, and metastasis. Cancer Metastasis Rev..

[11] Bellahcène A, Castronovo V, Ogbureke KU (2008). Small integrin-binding ligand N-linked glycoproteins (SIBLINGs): multifunctional proteins in cancer. Nat Rev Cancer.

[12] Roberts DD (2008). Thrombospondins: from structure to therapeutics. Cell Mol Life Sci.

[13] Lawler J (2002). Thrombospondin-1 as an endogenous inhibitor of angiogenesis and tumor growth. J Cell Mol Med..

[14] Yee KO, Streit M, Hawighorst T (2004). Expression of the type-1 repeats of thrombospondin-1 inhibits tumor growth through activation of transforming growth factor-beta. Am J Pathol.

[15] Kazerounian S, Yee KO, Lawler J (2008). Thrombospondins in cancer. Cell Mol Life Sci..

[16] Martin-Manso G, Galli S, Ridnour LA (2008). Thrombospondin 1 promotes tumor macrophage recruitment and enhances tumor cell cytotoxicity of differentiated U937 cells. Cancer Res..

[17] Young MF, Kerr JM, Termine JD (1990). cDNA cloning, mRNA distribution and heterogeneity, chromosomal location, and RFLP analysis of human osteopontin (OPN). Genomics..

[18] Anborgh PH, Mutrie JC, Tuck AB (2011). Pre- and post-translational regulation of osteopontin in cancer. J Cell Commun. Signal..

[19] Anborgh PH, Mutrie JC, Tuck AB (2010). Role of the metastasis-promoting protein osteopontin in the tumour microenvironment. J Cell Mol Med..

[20] El-Tanani MK (2008). Role of osteopontin in cellular signaling and metastatic phenotype. Front Biosc..

[21] Fedarko NS, Jain A, Karadag A (2001). Elevated serum bone sialoprotein and osteopontin in colon, breast, prostate, and lung cancer. Clin Cancer Res..

[22] Rud AK, Boye K, Øijordsbakken M (2013). Osteopontin is a prognostic biomarker in non-small cell lung cancer. BMC Cancer.

[23] Ostheimer C, Bache M, Güttler A (2014). Prognostic information of serial plasma osteopontin measurement in radiotherapy of non-small-cell lung cancer. BMC Cancer..

[24] Ahmed M, Behera R, Chakraborty G (2011). Osteopontin: a potentially important therapeutic target in cancer. Expert Opin Ther Targets.

[25] Isa S, Kawaguchi T, Teramukai S (2009). Serum osteopontin levels are highly prognostic for survival in advanced non–small-cell lung cancer: Results from JMTO LC 0004. J Thorac Oncol..

[26] Blasberg JD, Pass HI, Goparaju CM (2010). Reduction of elevated plasma osteopontin levels with resection of non-small-cell lung cancer. J Clin Oncol.

[27] Travis WD, Brambilla E, Muller-Hermlink HK (2004). Pathology and genetics of tumours of the lung, pleura, thymus and heart. World Health Organization classification of tumours.

[28] Travis WD, Brambilla E, Noguchi M (2011). International association for the study of lung cancer/american thoracic society/european respiratory society international multidisciplinary classification of lung adenocarcinoma. J Thorac Oncol.

[29] Goldstraw P, Crowley J, Chansky K (2007). The IASLC Lung Cancer Staging Project: proposals for the revision of the TNM stage groupings in the forthcoming (seventh) edition of the TNM Classification of malignant tumours. J Thorac Oncol.

[30] Pailler E, Adam J, Barthélémy A (2013). Detection of circulating tumor cells harboring a unique ALK rearrangement in ALK-positive non-small-cell lung cancer. J Clin Oncol.

[31] R Development Core Team. R: a language and environment for statistical computing. Vienna, Austria: R Foundation for Statistical Computing. http://www.rproject.org

[32] Psaila B, Lyden D (2009). The metastatic niche: adapting the foreign soil. Nat Rev Cancer..

[33] Chaffer CL, Weinberg RA (2011). A perspective on cancer cell metastasis. Science..

[34] Weber GF, Lett GS, Haubein NC (2010). Osteopontin is a marker for cancer aggressiveness and patient survival. Br J Cancer..

[35] Hu Z, Lin D, Yuan J (2005). Overexpression of osteopontin is associated with more aggressive phenotypes in human non-small cell lung cancer. Clin Cancer Res..

[36] Donati V, Boldrini L, Dell'Omodarme M (2005). Osteopontin expression and prognostic significance in non-small cell lung cancer. Clin Cancer Res..

[37] Le QT, Sutphin PD, Raychaudhuri S (2003). Identification of osteopontin as a prognostic plasma marker for head and neck squamous cell carcinomas. Clin Cancer Res..

[38] Rudland PS, Platt-Higgins A, El-Tanani M (2002). Prognostic significance of the metastasis- associated protein osteopontin in human breast cancer. Cancer Res..

[39] Agrawal D, Chen T, Irby R (2002). Osteopontin identified as lead marker of colon cancer progression, using pooled sample expression profiling. J Natl Cancer Inst..

[40] Mack PC, Redman MW, Chansky K (2008). Lower osteopontin plasma levels are associated with superior outcomes in advanced non-small-cell lung cancer patients receiving platinum-based chemotherapy: SWOG Study S0003. J Clin Oncol..

[41] Takenaka M, Hanagiri T, Shinohara S (2013). Serum level of osteopontin as a prognostic factor in patients who underwent surgical resection for non-small-cell lung cancer. Clin Lung Cancer.

[42] Yamaguchi M, Sugio K, Ondo K (2002). Reduced expression of thrombospondin-1 correlates with a poor prognosis in patients with non-small cell lung cancer. Lung Cancer.

[43] Grossfeld GD, Ginsberg DA, Stein JP (1997). Thrombospondin-1 expression in bladder cancer: association with p53 alterations, tumor angiogenesis, and tumor progression. J Natl Cancer Inst.

[44] Zabrenetzky V, Harris CC, Steeg PS (1994). Expression of the extracellular matrix molecule thrombospondin inversely correlates with malignant progression in melanoma, lung and breast carcinoma cell lines. Int J Cancer..

[45] Weinstat-Saslow DL, Zabrenetzky VS, VanHoutte K (1994). Transfection of thrombospondin 1 complementary DNA into a human breast carcinoma cell line reduces primary tumor growth, meta- static potential, and angiogenesis. Cancer Res..

