# Supplementary material for: Osteopontin and thrombospondin-1 play opposite roles in promoting tumor aggressiveness of primary resected non-small cell lung cancer
Source: BMC Cancer. 2016 Jul 15;16:483. doi: 10.1186/s12885-016-2541-5 (PMC4947364; doi:10.1186/s12885-016-2541-5)
Supplement: Additional file 1: Figure S1. — (A). Graphical representation of the ELISA data for OPN serum levels in healthy donors (n = 20) and population study (n = 171). Figure S1 (B). Graphical representation of the ELISA data for TSP-1 serum levels in healthy donors (n = 20) and population study (n = 171). (DOCX 154 kb) [file 12885_2016_2541_MOESM1_ESM.docx]

Additional file 1: Figure S1 (A). Graphical representation of the ELISA data for OPN serum levels in healthy donors (n=20) and population study (n=171).

Additional file 1: Figure S1 (B). Graphical representation of the ELISA data for TSP-1 serum levels in healthy donors (n=20) and population study (n=171).
